# Bacterial Spermosphere Inoculants Alter *N. benthamiana*-Plant Physiology and Host Bacterial Microbiome

**DOI:** 10.3390/plants13121677

**Published:** 2024-06-18

**Authors:** Andrea Sanchez Barrios, Derek Lundberg, Laura de Lorenzo, B Kirtley Amos, Meera Nair, Arthur Hunt, Seth DeBolt

**Affiliations:** 1 Department of Horticulture, University of Kentucky, Lexington, KY 40546, USA; andmari.sb@gmail.com (A.S.B.);; 2 Department of Molecular Biology, Max Planck Institute for Developmental Biology, 72076 Tübingen, Germany; derek.lundberg@gmail.com; 3 Department of Plant and Soil Sciences, University of Kentucky, Lexington, KY 40546, USA

**Keywords:** expansion, seed inoculum, microbiome, bacterial community, endophyte, spermosphere

## Abstract

In this study, we investigated the interplay between the spermosphere inoculum, host plant physiology, and endophytic compartment (EC) microbial community. Using 16S ribosomal RNA gene sequencing of root, stem, and leaf endophytic compartment communities, we established a baseline microbiome for *Nicotiana* sp. Phenotypic differences were observed due to the addition of some bacterial inoculants, correlated with endogenous auxin loads using transgenic plants expressing the auxin reporter pB-GFP::P87. When applied as spermosphere inoculants, select bacteria were found to create reproducible variation within the root EC microbiome and, more systematically, the host plant physiology. Our findings support the assertion that the spermosphere of plants is a zone that can influence the EC microbiome when applied in a greenhouse setting.

## 1. Introduction

The organization of microbial communities associated with a eukaryotic host is a common feature in terrestrial life [1] and is of particular importance in soil and agricultural settings [2,3]. Higher plants predominantly occupy a soil environment and are reliant on microbial functions to survive. This reliance for survival makes the study of their interactions an important research area. Plant–microbial associations have been deeply studied from multiple angles, categorizing them based on (A) bacterial communities that occur in the rhizosphere (soil root interface) [4,5], (B) bacterial communities that occur within the root itself [6,7,8,9], and (C) bacterial communities present within and outside of the phyllosphere (plant aerial tissue) [10,11,12,13]. These relationships can be considered endophytic, epiphytic, or closely associated. Each relationship can lead to different responses.

Endophytic microbial communities reside inside of plant tissues known as the endophytic compartment (EC) [7,9,14]. EC microbial communities have a significantly higher impact on the health, development, evolution, and chemical production of the plant when compared to microbial communities present only in the exterior tissue of the plant [15,16]. Microbial EC studies using culture-dependent techniques [10] have been shown to be limited in interpretation when trying to understand the composition of the microbiome. These studies were helpful in the isolation of some strains of bacteria due to their ability to alter normal plant physiology. Culture-independent sequencing techniques [8] were developed as a way to better define the existence and structure of the core root microbiome of whole plants [7,9,17] and individual organs in the plant. Amplification of a portion of the small-subunit ribosomal RNA gene (16S) sequencing has been adapted to exclude the abundant 16S sequences existing in the plant mitochondria and chloroplast [7]. This allows for a better representation of the phyla, family, and genera composition of bacteria and fungi inhabiting the plant.

Higher plants reside in a single location for their lifetime and utilize expansion-driven growth. Plant tissue expansion is regulated by the complex interplay of hormones that regulate specific features of the plant cell wall’s extensibility [18]. The controlled expansion of the plant cell wall constrains the internal cellular turgor pressure and facilities directional cellular expansion and plant morphogenesis. Particular microbes within the microbial community can have a direct or indirect functional association with a host plant to promote growth [19,20]. This occurrence poses several questions that remain underexplored. For instance, does the microbiome of a host plant remain constant or does the microbiome of the host plant respond to plant growth status, plant traits, or plant life history?

Deploying microbial inoculants as spermosphere adjuncts in agricultural settings [2] has been increasingly used for yield optimization [21], the suppression of pests [22,23], and the maintenance of fertility [24,25]. The introduction of an organism not familiar to the seed coat may result in numerous scenarios: for instance, (1) that organism may becomes dominant within the community, (2) the introduction of the new organism may cause a change or shock in the microbial community and further induce changes in the plants response, or (3) a combination of the two. Indeed, in prior domestication efforts, breeders have not focused on the microbial inoculation field, which is now emerging as an agricultural tool. Herein, we explored how adding a non-native bacterial inoculum to the sterilized Nicotiana spermosphere alters plant physiology and the plant EC microbiome. We paid attention to examining a highly domesticated versus a wild species in the Nicotiana genus. Phenotypic plant differences were observed due to the addition of the bacterial inoculum and linked to endogenous auxin loads. These results exemplify the importance of bacterial community composition near the spermosphere and how changes in that community can have effects on plant morphogenesis

## 2. Materials and Methods 

### 2.1. Soil Collection, Microbial Inoculum, Seed Selection, and Incubation Methods

Topsoil from the University of Kentucky North Farm, Lexington, Kentucky Spindletop Farm, (GPS coordinates: 38°07.555′ N, 84°30.901′ W) was collected, homogenized and mixed with perlite prior to use. Nutrient profiling of the soil was performed by core University of Kentucky Soil Regulatory Services (Table S1). *N. benthamiana*, *N. glutinosa*, *N. rustica*, and *N. tabacum* (two commercial lines were used, KY14 and TN90, as well as the *Nicotiana tabacum* L. cv. Samsun-NN, which is a well-described transgenic tobacco line to study abiotic stress [26,27,28]) seed was sourced from University of Kentucky Tobacco Research and Development Center (KTRDC). A germination cutoff of 90–100% was used. Surface sterilization of seeds was achieved via a 30% bleach rinse for 20 min followed by 70% ethanol rinse for one minute. These rinses were followed by three additional rinse cycles with sterile water. The sterilized material was tested by culturing on YPDA plates to ensure that no bacterial growth occurred. Bacterial library strains were sourced from the Switchgrass and Giant Burpee tomato library [29]. We screened 24 bacterial strains (Table S2) applied directly to seeds for growth promotion, growth suppression, or no influence. Strains were grown in YPD broth in medium flasks overnight (11 ± 2 h) at 28 °C on a rotary shaker. For inoculations, strains were grown at 28 °C on a rotary shaker until OD_600_ = 0.6. A total of 20 seeds were placed in each bacterial culture for spermosphere inoculation. Seeds were incubated in the culture for 12 h and kept in the rotary shaker/incubator.

### 2.2. Sample Selection and Processing

Morphological analyses followed the methods of Kelemu et al. [30] with some modification. Specifically, at two weeks, seedlings were checked for differences in the root system. After three to four weeks of inoculation, measurements were taken and recorded for traits including height, number of leaves (NL), leaf length (LL), leaf width (LW), and number of flowers. Measurements were repeated once a week for 60 days. Plants from both the treatment and control were harvested at approximately four to five weeks following the methodology developed by Lundberg et al. [9]. All plants were harvested, surface-sterilized, and processed on the same day.

### 2.3. DNA Extractions and Library Preparation

DNA extraction was performed using FASTDNA™-96 Soil Microbe DNA Kit (MP Biomedical, LLC, Santa Ana, CA, USA). Samples were previously separated based on being from the roots, stem, leaves, the rhizosphere, and outside the rhizosphere. All samples were placed in the freeze-dryer before being pulverized. Daisy bb gun beads were used to pre-pulverize the samples allowing stem and root samples to homogenize for optimal DNA isolation. After extraction, libraries were prepared following the protocol established by Lundberg et al., [31], where peptide nucleic acid (PNA) for mitochondrial (5′-GGCAAGTGTTCTTCGGA-3′) and plastid (5′-GGCTCAACCCTGGACAG-3′) rRNA and plastid sequences were used as elongation arrest clamps to prevent amplification of plant ribosomal 16S sequences.

### 2.4. PCR Quantification and Sequencing

The DNA concentrations of the final reactions obtained from the PCR step were measured in a 96-well plate format using PicoGreen fluorescent dye (Invitrogen, Waltham, MA, USA). This dye measures double-stranded DNA quantification and it was measured in a fluorescent plate reader (475 nm to 530 nm). After quantifying the amounts of DNA present, we analyzed a portion of the samples in an 1.5% agarose gel to ensure the presence of bands with a size of approximately 448 bp. The pooling of all samples from the prepared library was performed using equimolar ratios and samples were cleaned using AMPure beads at a 0.7:1 ratio. These samples were later eluted in 20 µL of deionized water to be denatured and loaded in the MiSeq machine by following the Illumina protocol and the standards established previously [31].

### 2.5. Demultiplexing and Heatmap Generation

After sequencing, the reads obtained from the Illumina platform were demultiplexed utilizing CASAVA software from Illumina (version1.8.2). A FASTA file was generated in which all the consensus sequences obtained per sample were stored. The software utilized was the Molecular Tag Toolbox 4.1.2. (MT-Toolbox, Google sites) [32]. R scripts were generated based on Lundberg et al. [31] to generate a graphical display of the abundance of different microbial organisms present in the samples. Rarefaction values varied based on the type of heatmap that was generated and were sorted into family, phylum, or operational taxonomic unit (OTU).

### 2.6. Phylum Analysis and Abundance for Genotype and Inoculation

Only the non-plant reads were classified at the phylum level. Reads from the same phyla were pooled, normalized and converted to a ratio by dividing the reads from each phylum by the total number of phylum-classifiable reads in that sample. For better visualization, those phyla representing less than 5% of the total in any sample were reclassified as “low abundance”. Data were plotted in R using the “Hist” function of ggplot2 (R Foundation for statistical computing, Vienna, Austria).

### 2.7. CAPSCALE Analysis

A constrained ordination routine analysis was used to determine if samples could be separated based on the treatment to which they were exposed. These analyses used a distance matrix between samples, showing the coordinates of each sample determined by the profile of OTU counts for that sample using the Bray–Curtis distance. The R packages used for the analysis were vegan, for capscale, ordination, and pscl (R foundation for statistical computing, Vienna, Austria).

### 2.8. Poly(A) Tag Library Preparation and Sequencing

Total RNA was isolated from *N. benthamiana* plants of approximately four to five weeks of age using the RNAeasy kit (Qiagen, Hilden, Germany). *Nicotiana* poly(A) tags (PATs) were generated with 1 µg of total RNA using Method B1 described in [33]. The resulting PATs were sequenced on an Illumina high-throughput sequencing DNA platform. In all cases, three independent biological replicates were used. The sequenced PAT-seq reads were processed using the pipeline as detailed in [33]. Briefly, sequences were demultiplexed and trimmed to remove the oligo-dT tracts and sequencing adapters. The processed tags were then mapped to the Nicotiana reference genome. The mapping output was saved in bam file format and used with BEDTools to determine the total count of PATs that mapped to individual annotated genes. The gene expression was determined using the empirical analysis of EDGE tool in CLC Genomics Workbench. Genes were considered significantly different using a *p*-value < 0.01 and a two-fold change. A total of three replicates were used per treatment.

### 2.9. CARD-FISH in Root Tissue

Briefly, roots from plants of 10- and 21-day old seedlings were collected and used for assessments. These were fixed and prepared as per Lebeis et al. [14]. Probes (EUB338 (59-GCTGCCTCCCGTAGGAGT-39, 35% formamide) were provided by the Lebeis lab at the University of Tennessee, Knoxville, which were selected using probeBase38 (http://www.microbial-ecology.net/default.asp, accessed on 4 January 2015) and labelled with enzyme horseradish peroxidase on the 5′ end (Invitrogen). Sample hybridization was performed as per [14]. For samples belonging to the 21-day-old group, we analyzed multiple panels from the transition zone to the tip and through the expansion zone. A similar approach was used for 10-day-old samples, but a full mosaic was built out of multiple sections of images.

### 2.10. Cold Treatment for Inoculants

All vials containing seeds and the inoculum that had reached OD_600_ = 0.6 were kept at 4 °C for 12 h in a rotary shaker. These were transferred to room temperature and shaken for an additional two hours prior to being placed in soil. All plants were grown in a greenhouse environment with the same set up mentioned in the prior method of inoculation.

### 2.11. Statistics for Morphological Data Analyses

All data collected were analyzed with SAS using GLM to generate means for each trait. We used Tukey’s test to separate means using a *p*-value of *p* < 0.05. Boxplots graphs were generated using BoxPlotR: a web-tool for the generation of box plots, created by the Tyers and Rappsilber labs (http://shiny.chemgrid.org/boxplotr/, accessed on 4 January 2015). Sample size was represented by the width of each box, and notches represented a 95% confidence between median differences. Tukey’s test was used to define the whiskers for each group sample. The number of samples was thirty-six total per year/per trait/per treatment (12 samples in each season per treatment).

### 2.12. Auxin Expression Related to Bacterial Treatments

Transgenic *N. tobacum* plants expressing a stable form of pB-GFP::P87 [34] obtained from the Maiti lab at the Kentucky Tobacco Research and Development Center (KTRDC) were PCR-verified for the presence of GFP. All seeds of *N. tobacum* pB-GFP::P87 were inoculated with Bs, Bc, Ms, or a mock control (media only) using the same protocol as described for *N. benthamiana*. IAA and 2,4-D treatment was performed by placing surface-sterilized seeds in plates containing two concentrations of IAA (1 uM and 10 uM) and 2,4-D (100 nM) that were added to the Murashige and Skoog growth medium used. Seeds were plated in Murashige and Skoog medium, and after 3 days post germination, they were visualized using a laser scanning confocal microscope (Olympus IX83). A total of 10 replicates per treatment were analyzed. The data obtained were analyzed using a Dunnett’s multiple comparison test against the control with a *p* < 0.05.

### 2.13. Hormone Profile

Auxin (IAA Indole-3-acetic acid), IAA-Asp N-(Indole-3-yl-acetyl)-aspartic acid, IAA-Glu N-(Indole-3-yl-acetyl)-glutamic acid, IAA-Ala N-(Indole-3-yl-acetyl)-alanine, IAA-Leu N-(Indole-3-yl-acetyl)-leucine, and IBA (Indole-3-butyric acid) were measured by means of HPLC-ESI-MS/MS following Zaharia et al., [35] and Lulsdorf et al. [36] as a fee-for-service product by the National Research Council of Canada. Conjugated forms of IAA were measured in addition to the free IAA because free IAA can represent around 25% of the total pool and conjugated forms create sinks of available IAA in situ [37].

### 2.14. Biochemical Test for Indole Production from Bacteria

This method was followed as described in Peterson and McLaughlin [38].

## 3. Results

### 3.1. Bacterial Inoculation Screening and Studies

**Figure 1:**
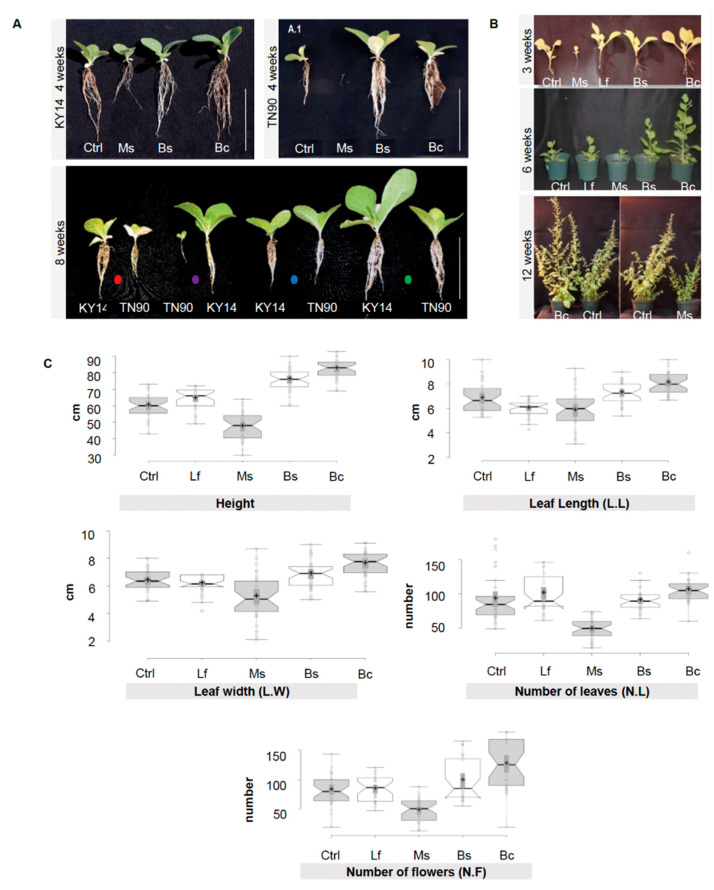
Morphological differences between *Nicotiana tabacum* and *Nicotiana benthamiana* treated with spermosphere bacterial inoculants. (**A**) *Nicotiana tabacum* line KY14 and TN90 at four weeks of growth. Plants at eight weeks of growth (side by side comparison of all treatments per tobacco line). Dots represent different bacterial spermosphere inoculants: red: control; purple: Ms; blue: Bs; and green: Bc. Scale bar = 10 cm. Lf was excluded to better illustrate the phenotypes and was not different to the control. (**B**) *N. benthamiana* control, Ms-, Lf-, Bs-, and Bc-treated plants grown in a greenhouse after three weeks show differences in morphology and root architecture. Plants at six weeks display a more distinct phenotype. Bc and Bs plants display the most growth and Ms treatments display the smallest phenotype. At 12 weeks, plants still show a distinctive phenotype. (**C**) *N. benthamiana* control, Ms-, Lf-, Bs-, and Bc-treated plants grown in a greenhouse over a year (composite of Spring, late Summer, and Fall/Winter 2013 data). Measurements of height, leaf length (L.L), leaf width (L.W), number of leaves (N.L), and number of flowers (N.F) were taken at between 28 and 35 days post germination, synchronized between treatments for each measurement. Means were separated using Tukey’s test; notches represent a significant difference among treatments at *p* < 0.05. n = 36 per trait/per year.

A library of 1000 EC microorganisms previously isolated from Switchgrass (*Panicum virgatum*) and Giant Burpee tomato (*Solanum lycopersicum* L.) [29,39] were screened, revealing 24 bacterial strains which induced a reproducible activation or suppression of growth (Table S2). From these 24 strains, we selected four bacterial inoculants that were used in the following experiments. The microorganisms selected were *Micrococcus* sp. (Ms), which induced growth suppression, and *Lysinibacillus fusiformis* (Lf), which had no growth influence. Two growth-promoting strains were identified from the screen: (1) *Bacilli*, *B.* sp. (Bs) and (2) *B. cereus* (Bc) (Figure 1A–C, Figures S1A,B, and S2). The *N. benthamiana* response to the four target microorganisms was reproducible over several generations and a range of conditions (3 years, 12 replicates per time point, changes in phenotypic traits recorded at 3, 6, and 12 weeks) (Figure 1, n = 36/phenotype/year; Figure S1).

### 3.2. Selection Based on the Capacity for the Seed–Inoculum Mixture to Alter Endogenous Auxin Levels

**Figure 2:**
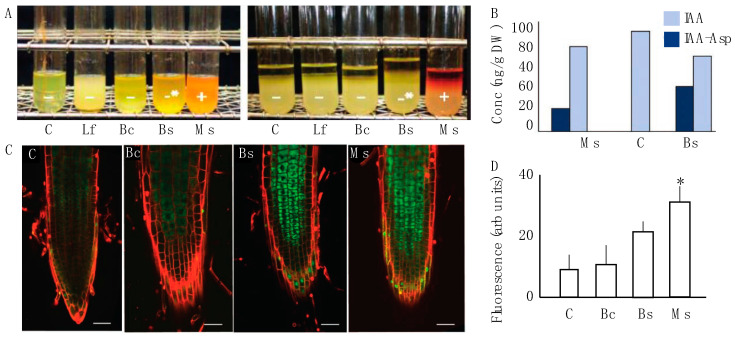
Auxin interaction between the microbial inoculum and host plant. (**A**) The indole test was performed for four bacterial spermosphere inoculants. Control and Lf showed a negative response. Bc and, to a greater degree, Bs had a modest positive response for an alternative indole product. Ms was the only treatment that displayed a positive production of indole which was visible with a red to orange hue. (**B**) Auxin metabolites were measured via gas chromatography in 10-day-old root tissue of two positive samples from A. Here, IAA-Asp was identified in the treatments Bc and Ms, but not the control (mock). (**C**) Laser scanning confocal micrographs of the fluorescence arising from GFP::P87 in *N. tabacum* seedling roots exposed to the control, Bc, Bs, and Ms treatments as the three samples that showed any indole response. Lf was excluded from this panel because it reduced the size of the micrographs and was not distinguishable from the control. A propidium iodide counter stain was used to highlight cell periphery and the green represents GFP::P87 signal. A total of 10 replicates for each treatment and the control were observed under a confocal microscope using a magnification of 200X. Scale bars represent 50 μm. (**D**) Fluorescence determination was performed for all treatments based on the analysis of total fluorescence in the root cap arising from GFP::P87. The only significantly increased P87 signal was observed for Ms treatment. Dunnett’s multiple comparison test against the water-control for each one suggests significance for Ms (*p* < 0.05), which is represented by asterisks.

The four selected bacterial strains were assayed for their ability to break down tryptophan and produce indole derivatives in vitro (Figure 2A). Using a microbial biochemical assay, we evaluated which strains produced indole breakdown products when exposed to Elrich’s reagent. A positive test resulted in visual hue shifts in the reagent mixture. Cultures corresponding to Lf, mock/control, and Bc were negative. Bs produced an intermediary positive response, which means that an alternative indole cleavage product like skatol could have been produced. The Ms bacterial cultures clearly revealed an ability to break down tryptophan to produce indole (Figure 2A). Following this result, we then performed HPLC-ESI-MS/MS on samples that distinctively had a phenotypic modification (Ms and Bc) and measured changes in biologically active IAA and IAA-Asp, an IAA conjugate with aspartic acid. The data showed that IAA was present in all samples, which was expected. However, IAA-Asp was found in Ms and Bc samples and not in the control (Figure 2B). As previously noted in the methods, the measurement of conjugated forms of auxin was performed due to only up to 25% of IAA pools in plants being free IAA, with conjugated forms comprising a sink of available IAA for physiological functions [37].

Based on the observed in vitro association between Ms, Bc, and auxin, we then evaluated the effect that strains had on the stimulation of auxin using the in planta biochemical auxin reporter P87 [34,40]. The results show differences in pB-GFP::P87 fluorescence in Bs and Ms roots compared to the control (Figure 2C), but these trends were only significantly different to the control for Ms. Quantitative assessment of pB-GFP::P87 fluorescence showed a significant increase 10 days after Ms application (*p* < 0.05, Bonferroni test). In the same timeframe, the Bs treatment induced a modest yet significant increase from the control (*p* > 0.05, Bonferroni test), but was significantly lower than Ms (*p* < 0.05) (Figure 2D Dunnett’s multiple comparison test). These data suggest that the Ms inoculum induced both in vitro and in vivo interactions with auxin biogenesis, which are functionally association with the mediation of plant expansion [18,41].

### 3.3. Functional Association between Inoculum and Transcript Abundance in the Host Plant

To complement the phenotypic responses between the host plant and the bacterial inoculant, the transcriptome of plants treated with each inoculant using the draft genome of *N. benthamiana* was cataloged [42]. These experiments compared transcriptional profile changes linked to each treatment. The draft genome of *N. benthamiana* is partially complete and patSeq tags were annotated against this incomplete genome. Firstly, experiments showed that approximately 75% of differentially expressed transcripts were commonly induced in plants treated with the growth-promoting Bs and Bc (fold change > 2, FDR *p*-value < 0.05). In contrast, only 20% of the induced transcriptome was shared between the growth-promoting Bs and -suppressing Ms (Figure S3A,B, Supplemental Dataset 5). The data broadly support a trend between the host phenotypic traits induced by an inoculum and the transcriptional output of the host plant.

### 3.4. A Baseline Bacterial Microbiome of Nicotiana Species

**Figure 3:**
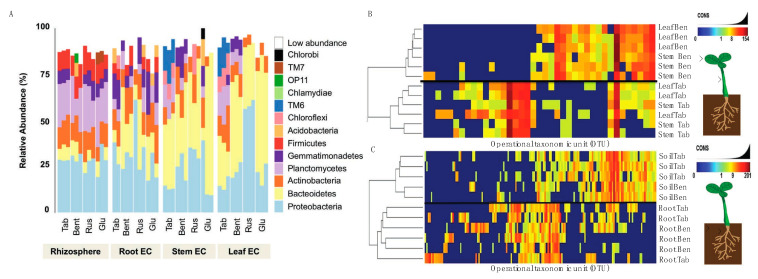
Establishment of the *Nicotiana* species microbiome using 16s rRNA sequencing. (**A**) Reads grouped at the phylum level had abundance differences for each phylum (represented as percentage) when compared between *Nicotiana tabacum*, *Nicotiana benthamiana*, *Nicotiana rustica*, and *Nicotiana glutinosa*. Samples with reads belonging to the phylum level that were less than 5% present were all classified under the “low abundance” category. Operation taxonomic unit (OTU) heatmaps were created for *Nicotiana tabacum* and *Nicotiana benthamiana* representing aboveground (**B**) and belowground (**C**) communities. Aboveground heat maps reveal a failure to separate by stem and leaf tissue, whereas belowground communities separated by soil and root. The presence and absence of selective OTU groups can be observed when comparing between aboveground and belowground bacterial communities.

Before inoculum experiments could be evaluated, we needed to generate the data for a *Nicotiana* bacterial microbiome. Here, three genotypes were selected including a domesticated commercial crop species, *N. tabacum* (KY14), as well as three wild varieties, *N. benthamiana*, *N. glutinosa* and *N. rustica*. The community assessments for the rhizosphere soil, root EC, stem EC, and leaf EC, were based on different tissues (Figure S4) and geographical origins (Figure S5). Community sequencing generated 1,491,297 merged paired-end consensus molecular-tagged reads across 92 samples (from which 36 belong to the genotype related-study). Bioinformatics’ removal of low-quality, plant-derived, and rare singleton sequences that did not cluster into operational taxonomic units (OTUs) of at least two sequences resulted in 318,860 reads (1225 reads and 243 OTUs per sample; Supplemental Dataset 1). The results reveal that the rhizosphere soil community was different from that of the root/stem/leaf EC (Figure 3A). Rhizosphere samples from all five genotypes showed similarities in abundance and the phyla present (Figure 3A), consistent with a core community. With these data, it is important to interpret the OTU data as linked to the methods of sequencing and identifying OTUs and may be an underrepresentation if sequence variation limited our ability to distinguish between overlapping taxa.

Bacterial communities of the stem and leaf EC (Figure 3A) differed from the root EC. General trends included a lower diversity of phyla and an increased abundance of *Bacterioidetes* in the stem and leaf EC compared with the root. A decreased abundance of *Proteobacteria*, *Plantomycetes*, *Firmicutes*, and *Actinobacteria* was also measured in stem and leaf EC compared with root. These phyla are abundant in soil [43,44,45,46] and were generally decreased in the stem and leaf EC of the species evaluated (Supplemental Dataset 2). It was found that *Chlorobi* and *Acidobacteria* were not widely distributed, found in one sample of *N. glutinosa* (Figure 3A). *Planctomycetes* and *Firmicutes* were more consistently present in the rhizosphere samples of all species compared with EC communities (Figure 3A, Supplemental Dataset 2). Collectively, these data showed a progression of community complexity from the rhizosphere soil (highest) to the roots to the stem and leaves (lowest).

From these data, *N. tabacum* and *N. benthamiana* were isolated to take a closer look at the OTU heat maps comparing aboveground tissues (Figure 3B) and belowground tissues (Figure 3C). One can generally observe these trends in Figure 3A, where the stem and leaf tissues of *N. tabacum* and *N. benthamiana* display different relative abundance between species but have similar community composition and abundance in both stem and leaves. It is to be expected that the rhizosphere (termed soil in Figure 3C) communities were nearly identical in abundance and composition when comparing *N. tabacum* and *N. benthamiana* because the same medium was used for growing both species. The only minor difference was the presence of *Acidobacteria* and *OP11* in the *N. benthamiana* rhizosphere (soil) community. Root communities were very similar and included *Proteobacteria*, *Bacterioidetes*, *Actinobacteria*, *Plactomycetes*, *Firmicutes*, *Gemmatimonadetes*, *Chloroflexi* and *Acidobacteria*.

### 3.5. Plants That Were Grown after Being Inoculated with a Single Microbial Species Displayed Specific Trends in the EC Community

A single-species inoculum was mixed with *Nicotiana* seeds (see methods for surfactant and density) and then the seed was allowed to germinate and grow in the medium. To understand how an inoculum changes the colonization process, we visualized the process using catalyzed reporter deposition and fluorescence in situ hybridization (CARD–FISH). CARD–FISH of whole root segments were imaged during two different time points, (1) after 10 days and (2) after 21 days. The reason for this timing is that we found that before 10 days old, the newly germinated seedlings were too immature to reliably image. The second time point provided a reliable gap for development and true leaf emergence. At the initial 10 day timepoint, the only samples that displayed increased CARD–FISH fluorescence were the inoculated plants (Figure S6). These were localized in the parenchymatic tissue, in the lateral roots and hairs. At the second 21-day timepoint, both the control and inoculated treatment routinely displayed CARD-FISH fluorescence, suggesting that colonization normalized between 10 and 21 days. These data provided a visual estimation that seed inoculation transiently affected the plant root EC community (Figure S6).

**Figure 4:**
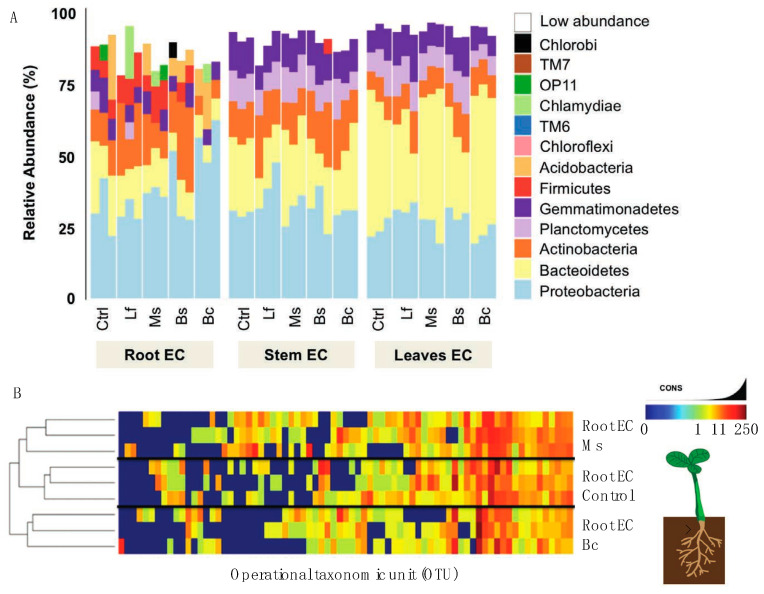
Interaction between microbial inoculum and the *Nicotiana* microbiome. (**A**) Reads grouped at the phyum level had abundance differences for each phylum (represented as percentage) when comparing *N. benthamiana* plants that had been exposed to bacterial inoculants as seed treatments. Samples with reads belonging to phylum level that were less than 5% present were all classified under the “low abundance” category. (**B**) Heatmap of bacterial OTUs present in samples from roots of treated and control plants. The treatments Bc and Ms were compared to control samples at the root level to show if, at a deeper classification, samples separated in composition.

**Figure 5:**
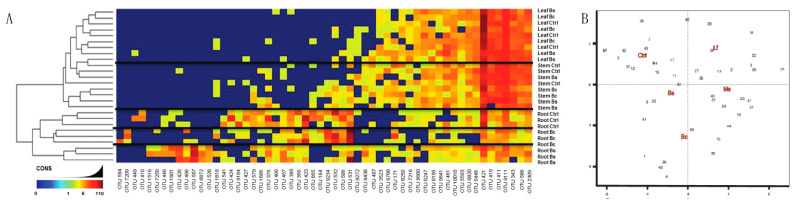
Similarities and differences by tissue, a community evaluation. (**A**) Heatmap of Bs and Bc plant organs compared to control samples at an OTU level. Sample organs separate when comparing root EC samples, but did not separate for aerial EC samples (stem and leaves). (**B**) Coordinates analysis (CAPSCALE analysis) of OTUs present in each treatment. For this, all OTUs from all treatments were used to build the comparison in which we were able to differentiate those treatments that were more closely related and those that were the most different. Data showed that Lf was similar to the control. In a similar manner, Bs was closely related to both the control and Bc, but different from Ms and Lf. Treatments like Bc and Ms clustered the farthest apart from control, consistent with microbial composition differences.

To further evaluate the influence of the seed inoculum on the plant microbiome, we adopted the same sequencing approach that we used for the microbiome/EC community composition. In terms of diversity and abundance of the EC community, at least four principal results were observed from the inoculation studies. Firstly, treatments and tissue-specific EC communities shared a core microbiome very similar (Figure 4A,B) to that which was observed in our genotype community description (Figure 3A). Secondly, we observed variations in the abundance of certain bacteria in inoculated plants. For example, *Acidobacteria* was present in low quantities in the control and Ms samples, but the abundance of this phylum increased among the Bs and Bc samples. Further, *OP11* was only found in control and Ms samples, and *Chlorobi* only in Bs samples (Figure 4A). When all tissue EC data were combined (Figure 5A) and a constrained analysis of principal coordinates was performed (Figure 5B), Bc and Ms accounted for the greatest proportion of variance in the data. Bc was a growth promoter and Ms was a growth suppressor. A third notable observation was that the Bc and Bs inoculations displayed an absence (Bc) or low abundance (Bs) of *Firmicutes* (Figure 4), revealing a significant drift from the core community in these treatments. A fourth result was that seed inoculants were seen to alter the root EC community more than was observed for the stem and leaf EC (Figure 4A,B), which is in line with the lower rarefaction used to generate the map comparisons (especially at OTU level) (Figure 5A). In stem and leaf EC, all treatments displayed a similar phylum composition, but the abundance of these classes differed. As in the reference microbiome, stem and leaf ECs were more restrictive of their EC inhabitance than roots (Supplemental Datasets 2 and 5).

### 3.6. Changing Temperatures during Spermosphere Association Was Important to the Host Response to Inoculation

The primary reason for this experiment was not to overinterpret this in relation to a complex ecological system but rather that a low temperature reduces metabolism and proliferation rate in the microbial organism as well as in the host plant. Here, we used the same inoculum described above, but before placing them in the soil, treatments (*N. benthamiana* seeds + inoculum) were exposed to a single low temperature (4 °C) overnight (12 h) before being brought back to room temperature for two hours and then placed into soil. The results were as follows: plants treated with Bc and Bs no longer exhibited growth promotion and were indistinguishable from control plants (Figure S7A). Ms-treated plants did not display a growth-suppressor phenotype to a modest growth promoter compared to the control. This trend was observed from the seedling stage to week seven (Figure S7B). To test whether phenotypic changes in the host had any association with the endogenous plant auxin levels that we previously observed, we inoculated pB-GFP::P87 *N. benthamiana* seeds with the abovementioned 4 °C overnight spermosphere treatment and evaluated fluorescence after 10 days. The data (Figure S8) indicated that the differences between endogenous auxin levels among treatments were no longer significant (n = 5, *p* > 0.05, Tukey–Kramer HSD test). These data suggest that a low-temperature environment that suppressed activity of the inoculum resulted in a loss of repeatability of the inoculum phenotypes.

## 4. Discussion

In order to begin to test the experimental concept of spermosphere associations, we first needed to establish a core microbiome for *Nicotiana* (Figures S1, S2 and Figure 3). The EC community of *Nicotiana* was comparable to other known plant microbiomes [14] and was based on a range of geographically diverse *Nicotiana* species. A notable result from these analyses was that the microbiome was influenced by domestication compared to wild relatives from South America and Australia (Supplemental Dataset 2), a result consistent with intense breeding for leaf volume and metabolites for smoking tobacco production. Interestingly, the general composition of our core microbiome displayed similarity to those reported by Saleem et al. [47] and Law et al. [48], despite these studies only employing an endophyte amplification approach in their commercial fields of *Nicotiana tabacum*. Our generated *Nicotiana* bacterial microbiome provided us with a baseline to compare inoculum treatments.

The spermosphere is a zone around the seed which separates the germinating embryo from the soil environment and is the first instance for exposure to the soil microbiome [49]. Since inoculants are applied to seeds, in situ imaging of the root EC community by CARD-FISH was performed. The data suggest that spermosphere inoculation caused a short-term increase in microbial signal in the root EC, which normalized between 10 and 21 days (Figure S6). However, by 21 days post inoculation, the treatment and control CARD-FISH signals normalized and became more spatially restricted around the stele tissue (Figure S6). The data suggest that heavily overrepresenting the inoculum of the plants during germination resulted in a short-term increase in early EC establishment, a phenomenon observed in microbiome associations with other complex eukaryotes [50,51].

In this study, the microbial associations in the spermosphere of greenhouse-grown *Nicotiana* plants correlated with tissue-specific variations in the EC (Figure 1, Figure 2 and Figure 3). As also noted in Song et al. [52], we observed that correlations between a changing inoculum and host traits are evident, but that the core microbiome remained largely intact. Herein, bacteria, when applied exogenously to the *Nicotiana* spermosphere, altered host plant morphogenesis and disrupted normal auxin physiology. Ms was the only treatment that displayed a positive production of indole. This auxin maxima was also observed by means of laser scanning confocal microscopy of the roots of transgenic *N. tobacum* plants expressing a GFP::P87 reporter (Figure 2). A way to interpret these data is that the in situ auxin maxima seen in the micrographs of Figure 2A caused a inhibition of expansion similar to endogenous auxin effects [53]. The Bs and Bc treatments cannot be explained in the same way, and this may be because they do not present a true endogenous auxin maxima at the site of root expansion. These are largely guilt by association pieces of evidence, and further work is needed to understand the interplay between EC production and the regulation of auxin in situ.

By design, we aimed to identify spermosphere associations that displayed a gradient of functional impact on the host (Figure 1). On one end of the scale, the inoculum Ms was capable of breaking down tryptophan to indole derivatives in vitro and perform the modulation of endogenous auxin levels in planta, resulting in consistently suppressed host expansion. On the other hand, the *Bacilli* sp. (Bs) inoculum was capable of inducing growth promotion in the host (Figure 1) consistent with other studies in specific *Firmicutes* [47]. Using a single genotype, we could then ask whether adding these spermosphere inoculum changed the EC community. The results show that the root EC community was most responsive to the spermosphere inoculum (Figure 3). As the site of the inoculum, this is not completely surprising. The changes did not strongly alter the core, but rather the distribution and abundance of members of the community. One exception to this was observed in the case of Bc and Bs treatments. Spermosphere inoculation with these *Bacilli* resulted in exclusion of the phylum *Firmicutes* from the root EC (Figure 4A). Given the replicated trial design, we do believe this to be a random observation. However, it was unclear to us what the mechanism for exclusion is. A possibility is that the alterations in the EC community are a result of altering host immunity due to the overrepresentation of one type of bacteria on the surface of the sterilized treated seeds [14]. Numerous Bs-specific transcripts were identified through PATseq assessment (Figure S4). Thorough investigation of these transcripts could yield gene targets that could be essential to establish healthy bacterial root EC communities. Again, further work is needed to study these associations, but one can envision numerous practical applications for harnessing plants’ immunity to exclude unwanted species or promote others.

Based on prior studies into the consistent nature of the core microbiome [7,51,54] and the response of the plant host [14,55,56,57], data supports the suggestion that plants can influence the selection of the EC and that the external microbial community can influence the plant. Our data are consistent with this postulation and suggest that the forces are not mutually exclusive and form something of a dynamic balance. These data are not unexpected given the wealth of information from multi-omics approaches [58]. Importantly, we simplified the experimental approaches by using a greenhouse medium substrate, a partially characterized inoculum series [29], and molecular approaches to examine associations. Using a visually obvious phenotype such as expansion (growth promotion and dwarfism) was particularly useful for making inferences. Associations with the hormone auxin were consistent with prior experiments [59], and while interesting, we cannot say if they are direct or indirect associations. As a major driver of plant form and function, these data could be useful. For instance, could an organism like Ms be applied to major grains crops to reduce height without a strong yield drag and lower the incidence of crop lodging?

The complications and environmental inconsistencies that have plagued efforts to field-validate microbial seed treatments compared with the consistency of agrochemicals are well documented [19,20,21,22,23,24,25,26,27,28]. An example that we observed was that varying the environmental temperature employed during seed inoculation caused reproducible and quite different phenotypic responses in the host plant (Figure S7). While agrochemicals also have environmental restrictions on their application times and conditions, these data highlight the need for additional experimentation before results can be taken from the relatively homogenous greenhouse to agricultural deployment.

## Data Availability

Data are contained within the article.

## Supplementary Materials

The following supporting information can be downloaded at: https://www.mdpi.com/article/10.3390/plants13121677/s1.
