# Supplementary material for: Bacterial Spermosphere Inoculants Alter N. benthamiana-Plant Physiology and Host Bacterial Microbiome
Source: Plants (Basel). 2024 Jun 18;13(12):1677. doi: 10.3390/plants13121677 (PMC11207711; doi:10.3390/plants13121677)
Supplement: Supplementary file 1 [file plants-13-01677-s001.zip › plants-2965130-supplementary.pdf]

Supplemental Table S1. Soil profile nutrients. Nutrient composition of soil collected from the University of Kentucky Spindletop Farm.

Lab #	Sample#	1M KCL soil pH	Calculated soil-water pH	Sikora II Buffer pH	P (lbs/a)	K (lbs/a)	Ca (lbs/a)	Mg (lbs/a)	Zn (lbs/a)
22889	1926	5.43	6.28	6.83	375	485	4018	360	9.1

Boron = 1.4 lb/ac, Meh3_Cu = 2.78 lb/ac, Meh3_Mn = 368 lb/ac, Meh3_Fe = 342 lb/ac,

Supplemental Table S2. Bacterial strains selected. Previously isolated bacterial organisms from plants grown under conventional and organic systems were sequenced for identification and archived in a bacterial and fungal library that belongs to the Debolt lab. All bacterial organisms mentioned in the list were assayed for their effect on plant development and growth.

Bacterial strains	Isolated from
Bulkholderia gladioli strain 33A	
Stenotrophomonas sp. 3c_5	
Microbacterium sp. Fek04	
Stenotrophomonas maltophilia strain H258	
***Microoccus sp.* HPABA07**	**Isolated from: Seed- Swicthgrass**
Chryseobacterium sp. JA37A1	
Bacillus cereus strain TT15	
Bacillus sp. TZQ2	
Paracocus sp. Zy-3	
Microbacterium oleivorans strain 1P06AB	
Bacillus thuringiensis srain DW-1T	
***Bacillus sp.* AS6**	**Isolated from: Stem- org. tomato**
Pseudomonas sp. SaCs17	
***Lysinibacillus fusiformis* strain Ba10**	**Isolated from: Leaf- Indian grass**
Lysinibacillus fusiformis strain R2	
Bacillus simplex strain X9	
Brevibacillus sp. Z0-YC6800	
Sphingomonas sp. PVS17	
***Bacillus cereus* strain EI-8**	**Isolated from: Stem- conv. tomato**
Denococcus sp. X-121	
Flavobacterium sp. CK18	
HQ324912.1 *Pseudomona putida* strain P-1017-1	
Bacillus cereus isolate T1-9	
Paenibacillus polymyxa strain SAZ2-6	

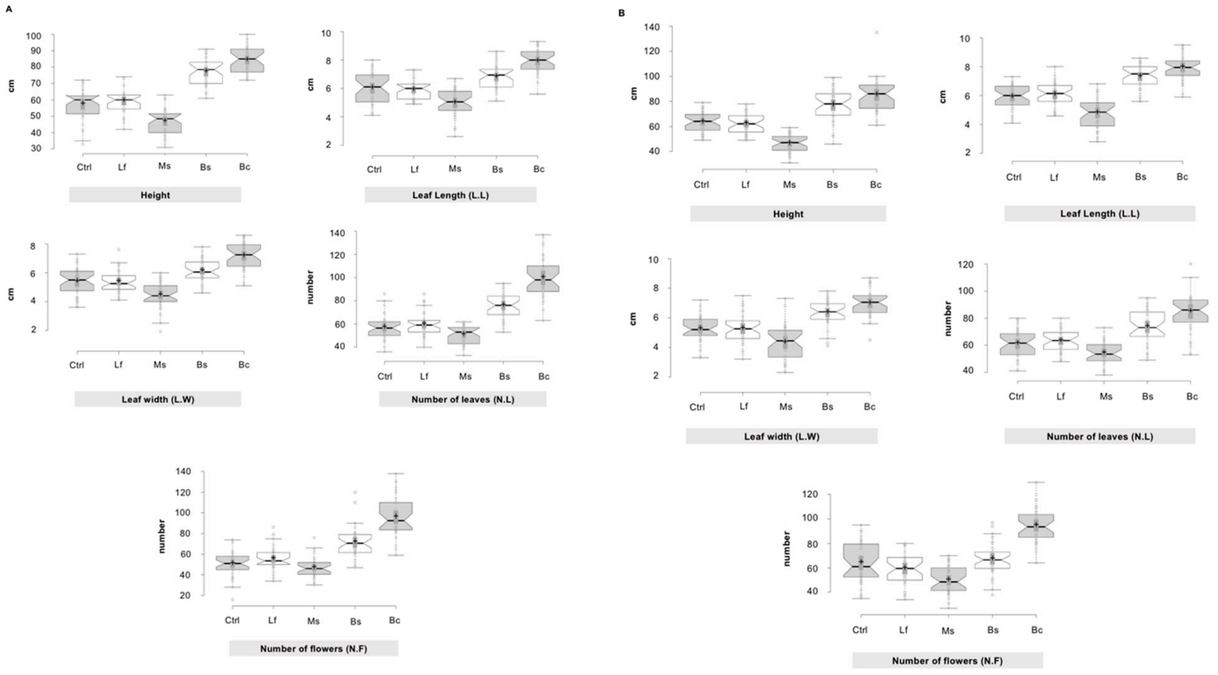

Figure S1. Morphological data of different traits evaluated in *Nicotiana benthamiana* plants. A) Control, Ms, Lf, Bs and Bc treated plants grown in the greenhouse during **the 2014 year** (composite of Spring, late Summer and Fall/Winter data) in 12 week old plants, and B) Control, Ms, Lf, Bs and Bc treated plants grown in greenhouse during the 2015 year (composite of Spring, late Summer and Fall/Winter data) in 12 week old plants. Measurements of Height, leaf length (L.L), leaf width (L.W), number of leaves (N.L), and number of flowers (N.F). Means were separated using Tukey's test, notches represent a significant difference among treatments ($p < 0.05$).

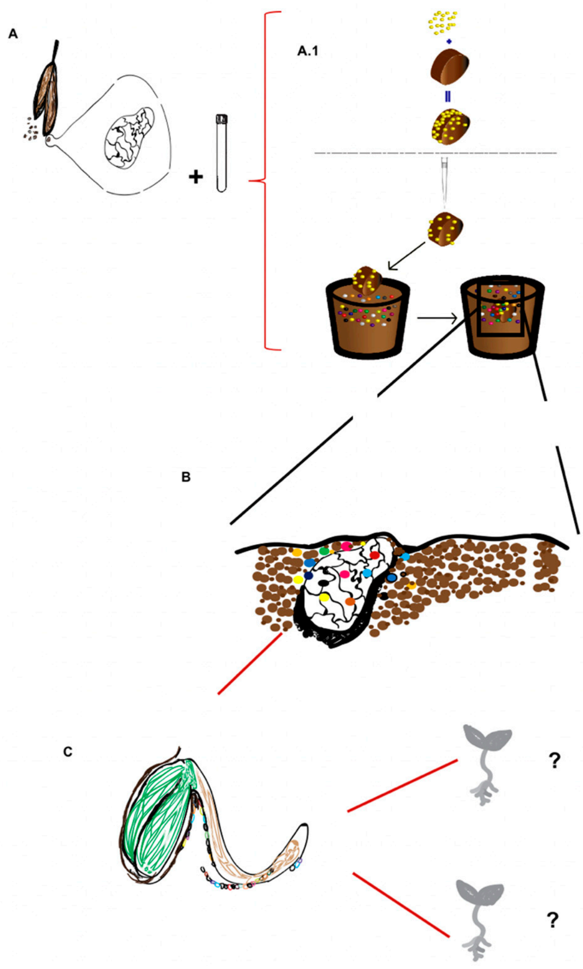

Figure S2. Diagram of the inoculation and screening process. A) Seeds (previously tested for germination percentage) were surface sterilized and co-culture with the bacterial strain used as an inoculum (monoculture). A.1) inoculation of seeds process: seeds are sterile and only colonize by the bacteria of interest. The overrepresented bacterial organism is expected to influence a selection from the soil base on an abnormal amount of bacterial cells of one specific organism. B) Spermosphere colonization by native bacteria from the soil (can vary based on the inoculum used) leads C) to a seedling developed with a first microbiome (from the spermosphere bacterial community) could have a response to it by developing a phenotype that could be positive (growth promoting) or negative (growth restrictive).

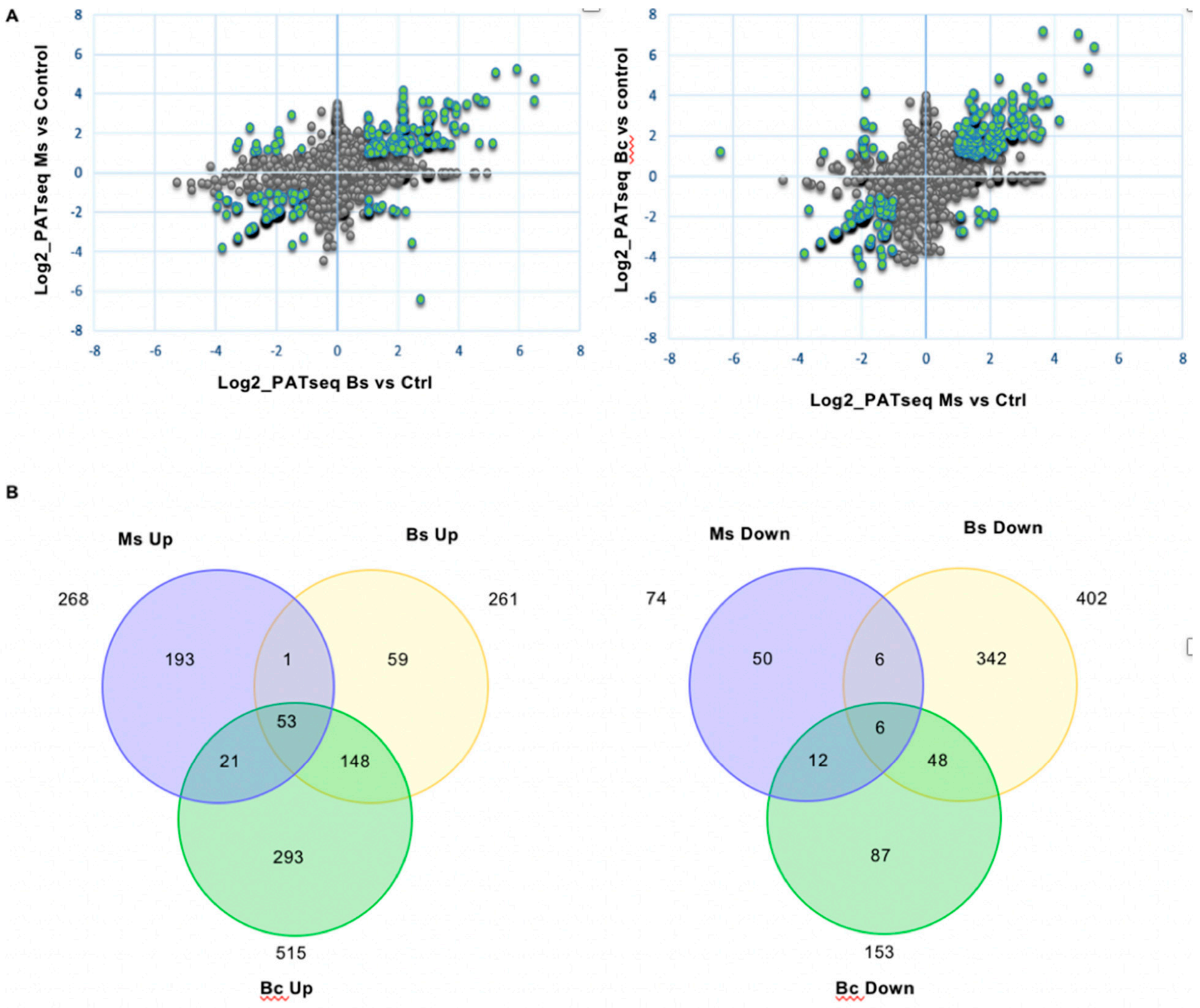

Figure S3. Comparison of the gene expression patterns of N. benthamiana after treatment with inoculations with Ms and Bc. A) Scatter plot comparing gene expression results from plants grown with Ms and Bc inoculum using PATseq. B) Overlapping genes grouped among all selected genes for each treatment.

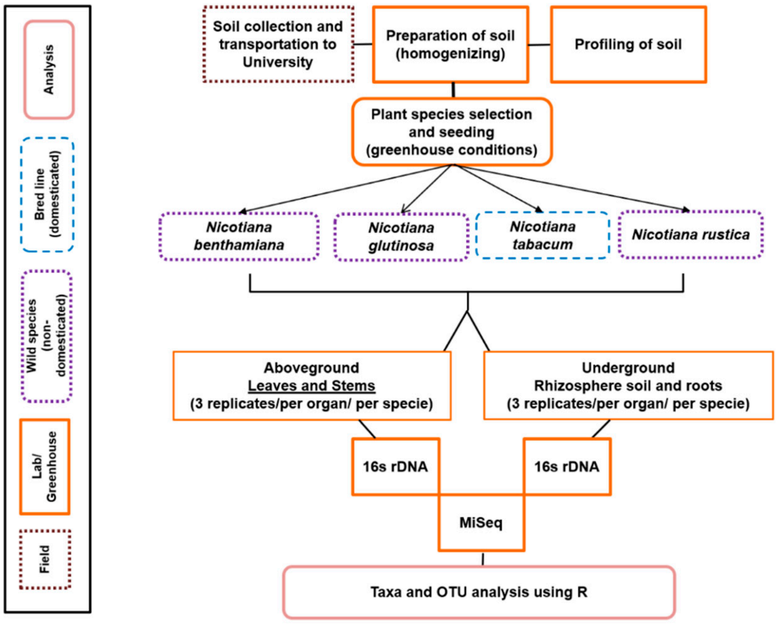

Figure S4. Community assessment workflow based on different tissues and genotypes.

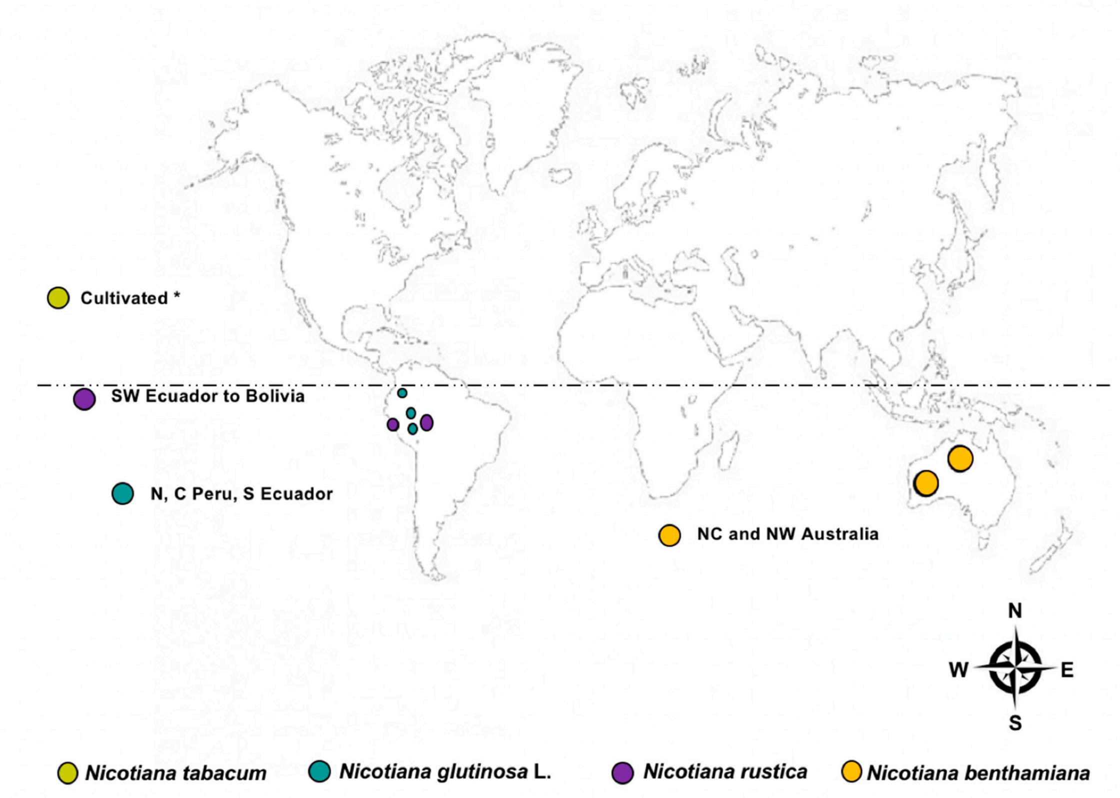

Figure S5. Geographical distribution of genotypes used to build a core microbiome of the *Nicotiana* genus. All seed used came from plants grown in the United States but the evolutionary origin of the seed spans different continents. Colored dots represent the different species selected. **Nicotiana tobacum* provenance is established by the place where it is cultivated and the original parental lines used.

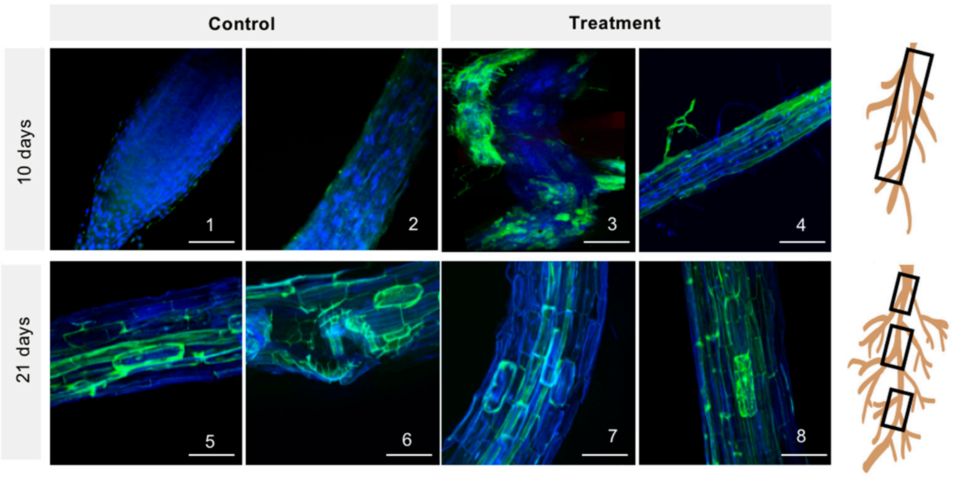

Supplemental Figure. S6. CARD-FISH imaging in treated and control plants using a eubacteria probe to determine localization of bacteria at different time points during growth and development. 1-2 & 3-4) Control at 10 days and 3 weeks, 5-6 & 7-8) Bs & Bc plants at 10 days and 3 weeks. At 10 days, most of the fluorescence is observed in the secondary roots and root hairs, at 3 weeks it can be observed mostly in the vascular system.

A

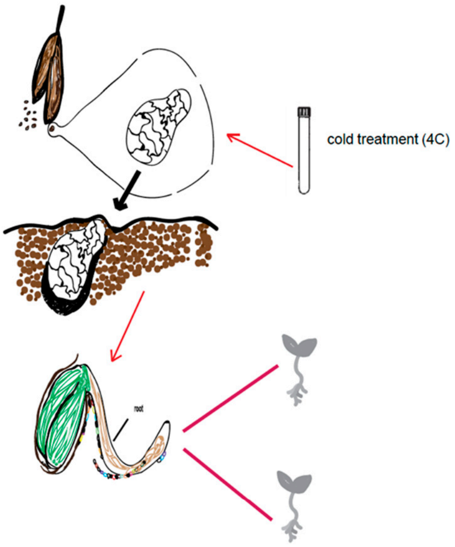

B

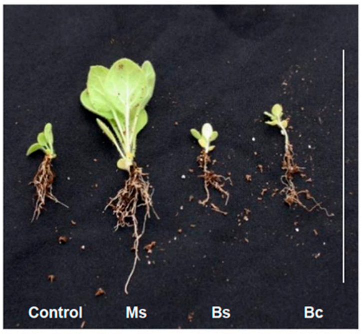

Figure S7. Schematic representation of the our spermosphere inoculum (A) experiment and resulting phenotypes upon exposure of the microbial inoculum to 4°C overnight (B). Plants were grown from seedling stage to week 7 and phenotypes are displayed visually (see methods). Scale bar = 10 cm.

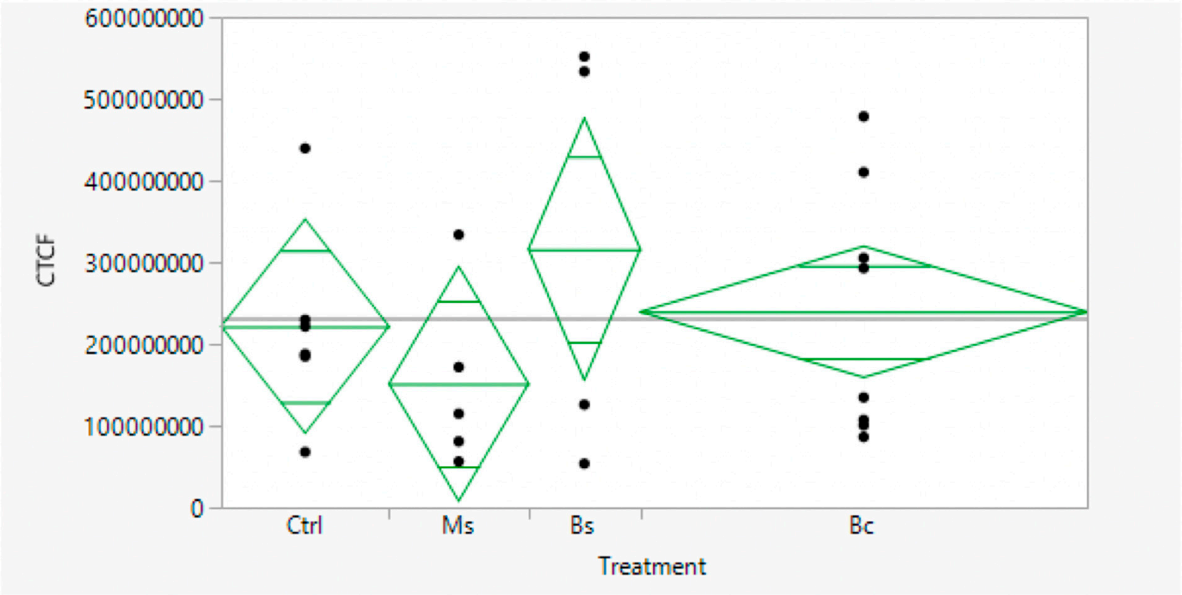

Figure S8. Corrected total cell fluorescence with means diamonds throughout different bacterial treatments exposed to 4°C for 12 hours. *Nicotiana tobacum* plants expressing DR5:GFP were imaged on an Olympus Fluorvew 1200 at 10x and corrected total cell fluorescence was calculated. A Dunnetts, Student T and Tukey-Kramer HSD test were performed in the JMP Pro, yielding no significance within any test.
